# Development of a screening strategy for new modulators of T cell receptor signaling and T cell activation

**DOI:** 10.1038/s41598-018-28106-5

**Published:** 2018-07-03

**Authors:** Elijah W. Chen, Joanna Brzostek, Nicholas R. J. Gascoigne, Vasily Rybakin

**Affiliations:** 1 Department of Microbiology and Immunology, Yong Loo Lin School of Medicine, National University of Singapore, 5 Science Drive 2 Blk MD4, Singapore, 117545 Singapore; 2 Department of Immunobiology, Rega Institute for Medical Research, KU Leuven, Herestraat 49, 3000 Leuven, Belgium

## Abstract

Activation of the T cell receptor (TCR) leads to the generation of a network of signaling events critical to the developmental decision making and activation of T cells. Various experimental approaches continue to identify new signaling molecules, adaptor proteins, and other regulators of TCR signaling. We propose a screening strategy for the identification of small molecules affecting TCR signaling based on the uncoupling of TCR stimulation from cellular responses in developing thymocytes. We demonstrate that this strategy successfully identifies inhibitors of kinases already shown to act downstream of TCR engagement, as well as new inhibitors. The proposed strategy is easily scalable for high throughput screening and will contribute to the identification of new druggable targets in T cell activation.

## Introduction

T cells are lymphocytes that express the T cell receptor (TCR), and they play an important role in adaptive immunity. Through the TCR, T cells are able to recognise their ligands: a complex of a peptide on major histocompatibility complex (peptide-MHC), presented on antigen presenting cells (APCs). Cellular signaling downstream of the TCR is critical for the development and activation of T cells. In the thymus, stimulation by self peptide-MHC directs developmental decision making by immature T cells^1^. The selection process of T cells begins at the double positive (DP) stage in which the T cells express both the CD4 and CD8 co-receptors. In the periphery, non-self antigens drive activation and proliferation of mature T cells, whereas stimulation with self peptides remains important in the homeostasis of several T cell subsets, contributes to peripheral differentiation of helper T cells, and may provide tonic signaling required for T cell survival and homeostatic expansion^2,3^. TCR triggering elicits a highly complex signal transduction mechanism which involves multiple pathways originating from the “signalosome”, a signaling platform assembled in the vicinity of the activated receptor and acting as a scaffold for multiple signaling molecules^4^. Although the biochemistry of TCR signal transduction has been actively studied for over three decades, new components of TCR signaling machinery are being continuously discovered^5,6^.

Targeting TCR engagement and signal transduction is highly relevant to the clinic, particularly in the context of autoimmunity, where various strategies for interference with T cell activation, proliferation, and viability are considered as important therapeutic approaches^7^. Strategies for direct inhibition of TCR signaling are largely based on interference with protein kinase and phosphatase activity. For example, inhibition of protein kinases acting early in T cell receptor signaling, in particular that of Src family kinases, blocks T cell activation *in vitro* and *in vivo*^8–10^. Conversely, inhibition of tyrosine phosphatases potentiates T cell activation^11^ and is investigated as a tool to reinvigorate exhausted T cells in which increased phosphatase activity downstream of inhibitory receptors raises the threshold for TCR signal generation^12,13^. Inhibition of phosphatases to enhance T cell responses would also be a viable option for tumour immunotherapy. Dampening of T cell activation and autoimmune responses was also observed upon treatment with a new small molecule inhibitor of CD3ε binding to the adaptor protein Nck^14^. Multiple therapeutic compounds, such as non-steroid anti-inflammatory drugs, may affect components of TCR signal transduction machinery as an off-target effect and therefore interfere with T cell activation^15,16^.

We have previously devised a flow cytometry-based assay to investigate the responses of *ex vivo*-stimulated developing T cells to a range of peptide-MHC stimuli^17^. Because immature thymocytes initiate apoptotic programs in response to strong stimulation through the TCR, we incubated TCR-transgenic thymocytes with peptide-MHC tetramers of increasing potency and detected caspase activation as a readout for the cellular perception of the corresponding signals. Here, we adapt this assay for the screening of small molecule libraries. We chose to use a commercially available library of approximately 150 kinase inhibitors and used the method described above^17^ to investigate thymocyte responsiveness. We report a strategy to pre-screen the compounds of interest for potential interference with thymocyte viability in the absence of antigenic stimulation, and to screen TCR-polyclonal thymocytes pre-treated with inhibitors for the interruption of TCR signaling. We further demonstrate additional factors of interest that can be included to refine the assay. Our initial screen identified multiple compounds that inhibit kinases with well-established functions in the TCR cascade, as well as potential new druggable targets. Several compounds were selected for validation in peripheral T cells.

The proposed assay can be directly applied for the screening of comparatively small compound libraries and easily adapted for higher throughput screening.

## Materials and Methods

### Mice

Wild type C57BL/6 (B6) mice were bred in the animal facility under restricted flora conditions at National University of Singapore (Singapore) in accordance with IACUC guidelines. Thymocytes and lymphocytes were isolated from 6–8-week old male and female B6 mice. The thymi and lymph nodes of the mice were extracted from the mice, mashed using a sterile syringe, and carefully homogenized by passing through a 70 μm cell strainer. Cells were maintained in complete RPMI medium (Hyclone) supplemented with 10% fetal calf serum (Hyclone), 100 U/ml penicillin and 0.1 mg/ml streptomycin (Hyclone), 2 mM L-glutamate (Hyclone), 1 mM sodium pyruvate (Hyclone), 50 μM β-mercaptoethanol (Sigma-Aldrich). The authors confirm that all experiments were carried out in accordance with relevant guidelines and regulations, and that all experimental protocols were approved by the National University of Singapore Institutional Animal Care and Use Committee (protocol numbers 2013-04470, approved 23/08/2013, and 2017-00478, approved 19/07/2017).

### Flow cytometry and antibodies

BD LSRFortessa X-20 flow cytometer (BD Biosciences) and FlowJo software (Treestar) were used for acquisition and analysis, respectively. The antibodies used include anti-mouse CD8 (#563786), anti-mouse CD25 (#558642), anti-mouse TCRβ (#553174) from BD Biosciences; anti-mouse CD4 (#100544) from BioLegend; anti-mouse CD3 (#14-0031-86), anti-mouse CD4 (#17-0042-83), anti-mouse CD4 (#17-0041-83) and anti-mouse CD69 (#25-0691-82) from eBioscience. Kinase inhibitor library was from Cayman Chemical (#10505).

### Viability screen

Thymocytes from B6 mice were plated in 96-well U-bottom plates at 1 × 10^6^ cells per well in 250 μl of complete RPMI and incubated with the inhibitors in a 96-well U-bottom plate for 18 h. Cells were analyzed by flow cytometry. FSC and SSC plots were used to determine live cell population. Non-stimulated samples (positive gating) and 5 µM dexamethasone treated (Sigma-Aldrich; negative gating) samples were used to determine the gating for the live cell population. For the assessment of reproducibility, technical replicates of the controls were carried out with 8 replicates of each condition per plate. Cells were transferred into individual 12 × 75 mm polystyrene tubes (BD Falcon) prior to analysis by flow cytometry.

### Activation screens

Thymocytes from B6 mice were plated in 96-well U-bottom plates at 1 × 10^6^ cells per well in 250 μl of complete RPMI. Thymocytes were stimulated for 18 h with anti-CD3/CD28 magnetic beads (Gibco, #11452D), at a ratio of 1 bead to 2.5 cells. After stimulation, cells were surface-stained for CD4, CD8, TCRβ and CD69 for 30 min on ice, in 75 μl of the staining mix. Fixation and permeabilization, and intracellular staining for intracellular active caspase 3 were carried following the manufacturer’s protocol (BD Pharmingen, #550480). The staining procedures were carried out as described^17^. For the stimulation assays of peripheral lymphocytes, 96-well U-bottom plates were pre-coated with 75 μl of 2 μg/ml anti-CD3 antibody solution and incubated overnight at 4 °C. On the day of the stimulation assay, the anti-CD3 antibody solution was removed, and peripheral lymphocytes from B6 mice were then plated into the wells at 1 × 10^6^ cells per well in 250 μl of complete RPMI. For 3 h stimulation assays, cells were stained with surface antibodies for CD4, CD8, CD69 and TCRβ for 30 min on ice, in 75 μl of the staining mix. For 18 h stimulation assays, cells were stained with surface antibodies for CD4, CD8, CD25 and TCRβ. Cells were transferred into individual 12 × 75 mm polystyrene tubes prior to analysis by flow cytometry.

### Reproducibility screens

Stimulation and activation steps are the same as described above. For screens assessing the reproducibility of replicates within the same plate, 8 replicates were prepared for each condition. For screens assessing the reproducibility of the data between different plates, 4 replicates were prepared for each condition, and a total of 8 separate plates were prepared. Cells were stimulated, stained, and analyzed as above.

### Statistical analyses

Statistical analyses were carried out using GraphPad Prism (GraphPad Software). Coefficient of variation (CV), a ratio of the standard variation to the mean expressed as a percentage, was used to assess the reproducibility of the screening assays.

## Results and Discussion

### Thymocyte viability assay

**Figure 1:**
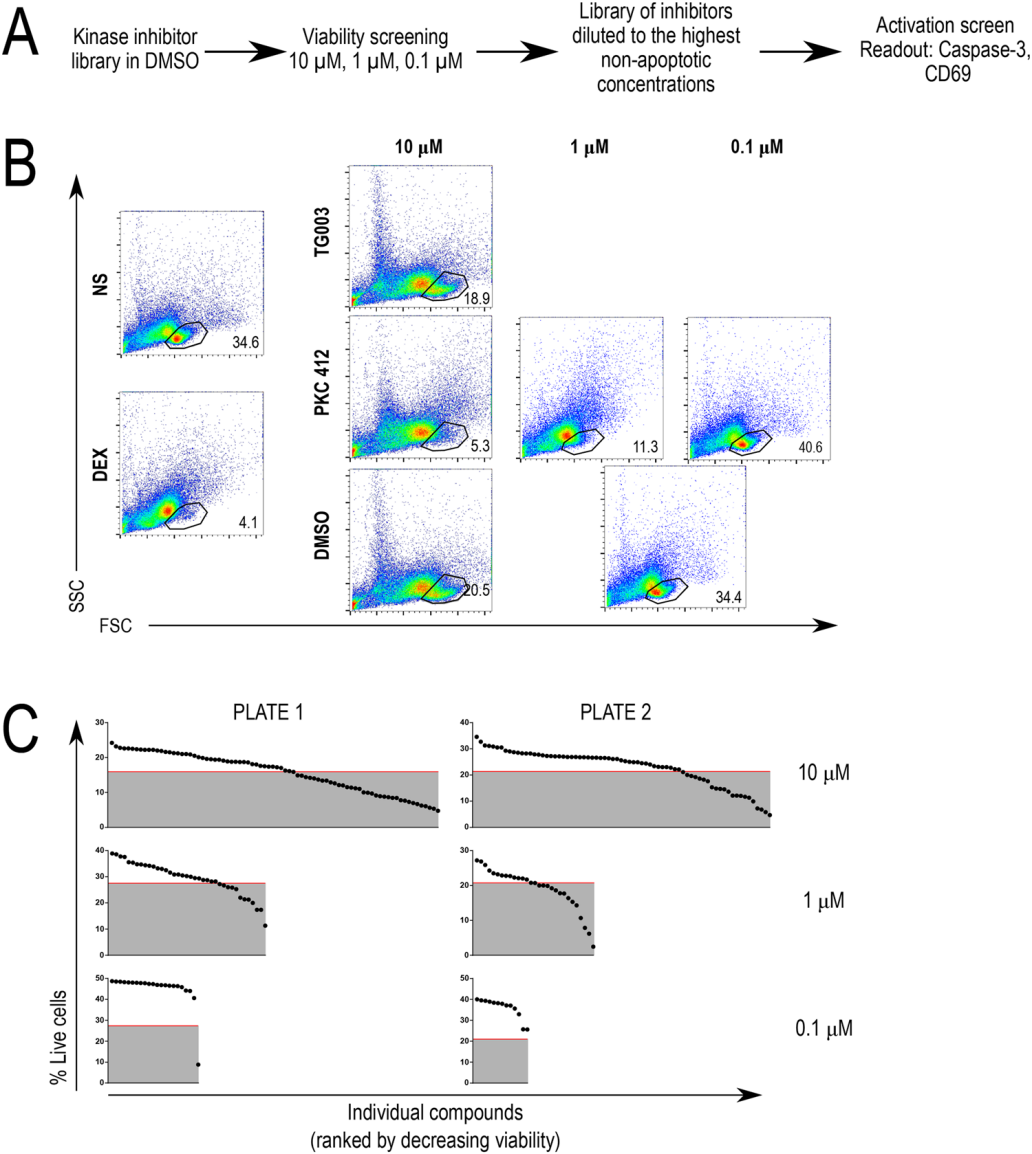
Experimental design and viability screening. (**A)** Schematic of the experimental design for the kinase library screening. A thymocyte viability screen was first carried out to determine the maximum concentration of the kinase inhibitors to be used. The final concentrations of the inhibitors follow a series of 10-fold dilutions: 10 μM, 1 μM, or 0.1 μM. **(B)** Gating strategy in viability assays. The live cell gate was created from the forward and side scatter plots as described^17^. Representative plots for the negative control (non-stimulated, NS) and positive control, (dexamethasone-treated, DEX) samples are shown to the left. To determine the highest non-toxic concentrations of inhibitors, the percentages of live cells were compared to DMSO-treated controls within the same plate. An empirically selected value of 80% of the percentage of live cells in the DMSO-treated samples was used as the upper cutoff for toxicity. Inhibitors that were deemed to be too toxic at the tested concentration were subject to further viability assays at 10-fold lower concentrations. Note the common negative control (DMSO-treated, DMSO) in the dilution experiment for the 1 μM and 0.1 μM samples. **(C)** Compete data set from the viability pre-screen. The red line represents the cut off value which corresponds to the 80th percentile of the percentage of live cells in DMSO-treated samples. Inhibitors falling below the red line were re-tested at lower concentrations as in 1C.

The overall logic of the proposed screening strategy is depicted in Fig. 1A. Because the primary readout of the screen is the reduction of apoptotic response in thymocytes stimulated by TCR crosslinking, it was essential to pre-screen the compounds of interest for their own effects on thymocyte viability in the absence of such TCR stimuli. Thymocytes cultured in the presence or absence of a proapoptotic agent dexamethasone at 5 μM were used as positive and negative controls for population gating in flow cytometry experiments (Fig. 1B, left panel). We started the pre-screen by applying all library compounds at the concentration of 10 μM to thymocytes and assaying their viability after an 18-hour incubation. Inhibitors that displayed >20% increase in cell death were subsequently tested at the concentrations of 1 μM and 0.1 μM (Fig. 1B,C). Figure 1B (right panel) shows the results of viability testing of two individual compounds. TG003 (1-(3-ethyl-5-methoxy-2(3 H)-benzothiazolylidene)-2-propanone; CAS 300801-52-9), an inhibitor of Clk, a Cdc2-like kinase^18^, showed negligible decrease in thymocyte viability at 10 μM concentration and was used at this concentration in functional screens. PKC 412 (N-[(9 S,10 R,11 R,13 R)-2,3,10,11,12,13-hexahydro-10-methoxy-9-methyl-1-oxo-9,13-epoxy-1H,9H-diindolo[1,2,3-gh:3′,2′,1′-lm]pyrrolo[3,4-j][1,7]benzodiazonin-11-yl]-N-methyl-benzamide; CAS 120685-11-2), a broad range kinase inhibitor^19^, strongly increased cell death at 10 μM and 1 μM concentrations but not at 0.1 μM, and therefore the latter concentration was used in functional screens. Only one compound, staurosporine (2,3,10,11,12,13-hexahydro-10R-methoxy-9S-methyl-11R-methylamino-9S,13R-epoxy-1H,9H-diindolo[1,2,3-gh;3′,2′,1′-lm]pyrrolo[3,4-j][1,7]benzodiazonin-1-one; CAS 62996-74-1), a broad range protein kinase inhibitor and known proapoptotic agent^20^, displayed a significant increase in thymocyte apoptosis at 0.1 μM (Fig. 1C; bottom left). Final concentrations selected for subsequent experiments were the highest concentrations that did not increase thymocyte apoptosis by more than 20%.

### Thymocyte activation assay

**Figure 2:**
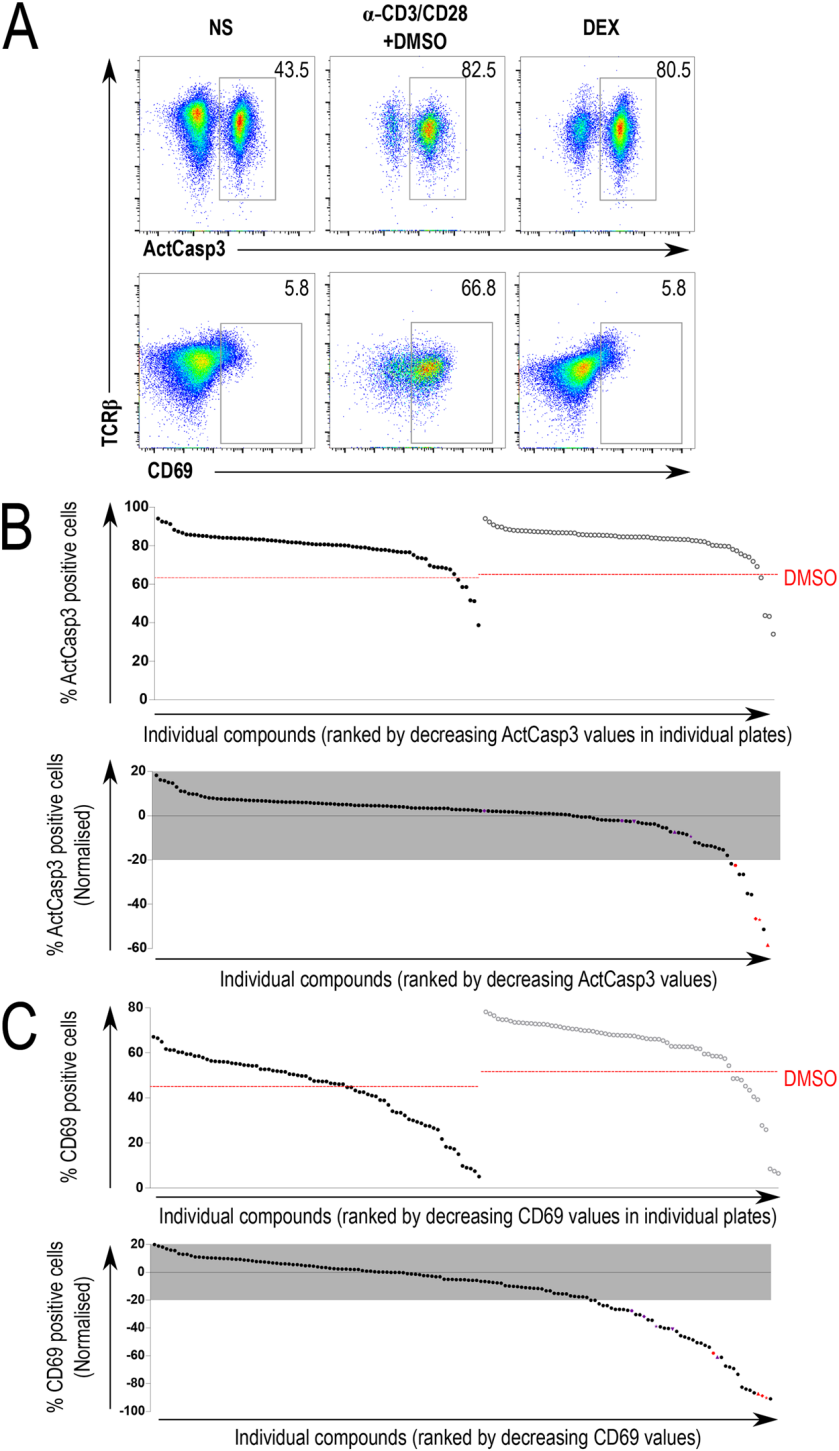
Functional screening of thymocyte activation. (**A)** Gating strategy and controls. Flow cytometry plots showing active caspase 3 (ActCasp3), CD69, and TCRβ staining within the thymocyte DP gate. Representative plots for each condition were selected from a set of quadruplicates. NS: Non-stimulated; α-CD3/CD28 + DMSO: Samples stimulated with CD3/CD28-coated beads and treated with DMSO; DEX: dexamethasone-treated samples. **(B,C)** Complete data from the functional screen. Thymocytes were stimulated with anti-CD3/CD28 beads for 18 h, surface stained for CD4, CD8 and CD69, permeabilized, and stained for intracellular active caspase 3. Graphs show the percentage of cells positive for active caspase 3 (ActCasp3; panel B) or CD69 expression (panel C), arranged in order of decreasing value. Raw values (top rows) and values normalized to controls (bottom rows) are shown separately for both active caspase 3 and CD69. The red lines in the raw data charts represent the average value of the DMSO-treated stimulated samples for each experiment. Normalized values are obtained by normalizing individual values to the DMSO-treated stimulated samples. An empirically selected window of ±20% is highlighted in grey in the normalized charts to represent the compounds that were filtered out. Colored symbols represent compounds detailed in Figs 3 and 4.

We then applied the inhibitors at the concentrations determined above to the TCR signal-induced thymocyte apoptosis assay. Cells were incubated in the presence of anti-CD3/CD28 beads and inhibitors for 18 h and assayed for caspase 3 activation using specific antibodies and flow cytometry-based detection in DP thymocytes^17^. As expected, strong TCR stimulation by antibody-mediated crosslinking elicited a response characterized by the activation of caspase 3 and upregulation of surface activation marker CD69 and TCR downregulation, whereas the TCR-independent induction of apoptosis by dexamethasone was not accompanied by CD69 upregulation or TCR internalization (Fig. 2A). A peculiar feature of this assay is that for inhibitors that suppress TCR signaling in the thymocytes, cell death is reduced, thereby accentuating the difference of TCR-independent effect of the inhibitors on cell death. Several tested substances inhibited the cellular response to CD3/CD28 stimulation, as seen by lower caspase activation and lower expression of CD69 (Fig. 2B,C). As seen from Fig. 2, several compounds affected both hallmarks of thymocyte activation, while some only inhibited CD69 upregulation or caspase 3 activity.

**Figure 3:**
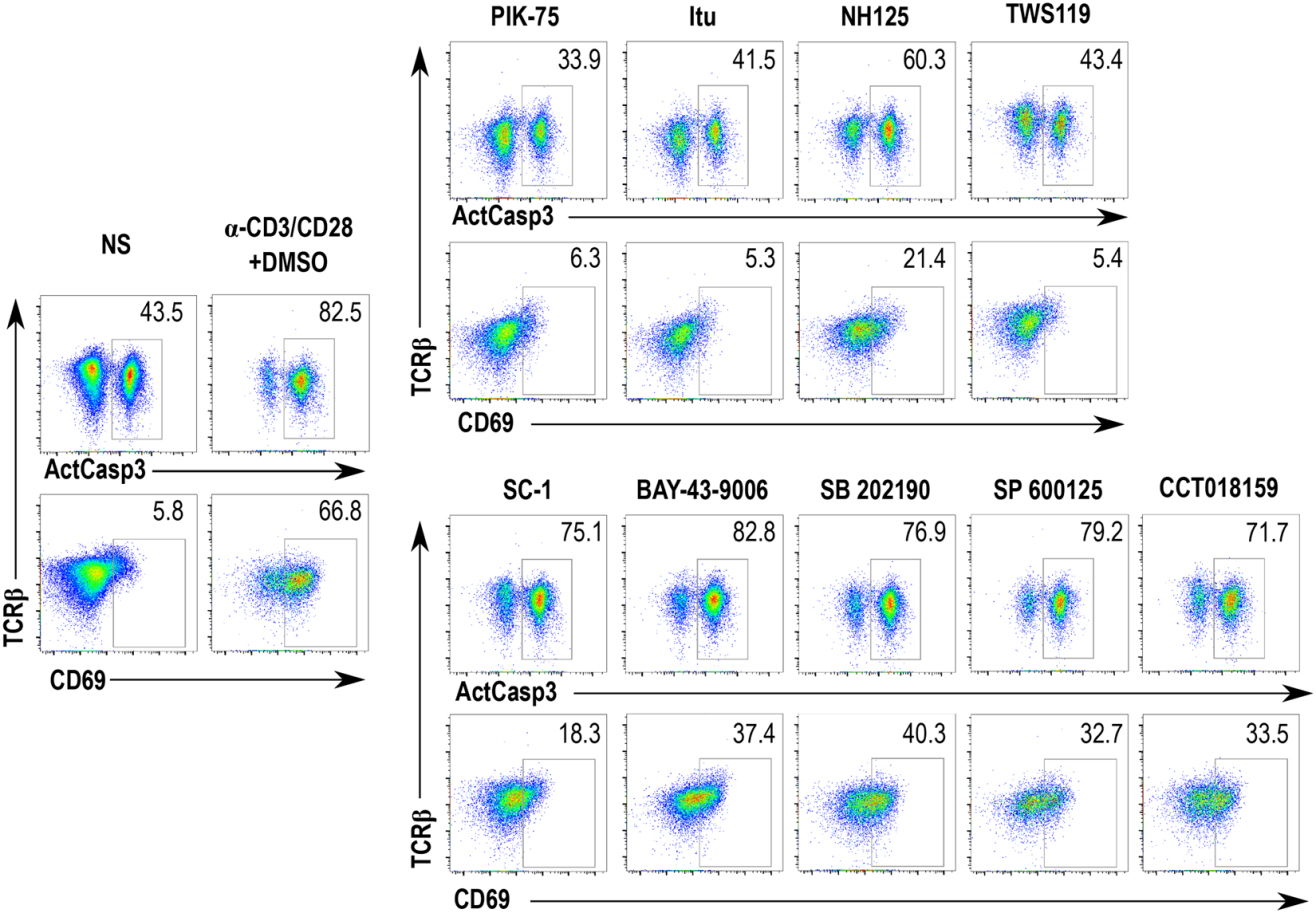
Identification of known and new inhibitors of thymocyte activation among positive hits from the functional screen. Flow cytometry plots of inhibitors that either suppressed both caspase 3 activation and CD69 upregulation (top panel) or only CD69 upregulation (bottom panel) in thymocytes stimulated and analyzed as depicted in Fig. 2. Representative plots of the controls (NS and α-CD3/CD28 + DMSO) shown here were selected from a set of quadruplicates. Individual compounds were tested individually without replicates. NS: Non-stimulated samples (negative control); α-CD3/CD28 + DMSO: samples stimulated in the presence of DMSO (positive control).

The screen yielded several expected hits, such as broad range kinase inhibitors and small molecules that inhibit enzymes known to act in the TCR pathway. Examples of several such compounds, which displayed different degrees of inhibition of thymocyte activation, are shown in Fig. 3, top panel. PIK-75 (2-methyl-5-nitro-2-[(6-bromoimidazo[1,2-a]pyridin-3-yl)methylene]-1-methylhydrazide-benzenesulfonic acid, monohydrochloride; CAS 372196-77-5) is an imidazopyridine compound that inhibits several isoforms of the phosphatidylinositol-3-kinase^21^. Itu (5-Iodotubercidin, 5-iodo-7-β-D-ribofuranosyl-7H-pyrrolo[2,3-d]pyrimidin-4-amine; CAS 24386-93-4) is an adenosine derivative that inhibits protein kinase C, protein kinase A, and several others^22^. NH-125 (1-hexadecyl-2-methyl-3-(phenylmethyl)-1H-imidazolium-iodide; CAS 278603-08-0) is another broad range inhibitor with activities on eEF-2K and multiple other kinases^23^.

Several compounds specifically blocked CD69 upregulation, but not caspase 3 activation (Fig. 3, bottom panel). Among these, we identified a RasGAP1 and Erk inhibitor Sc-1 (N-(3-(7-(1,3-dimethyl-1H-pyrazol-5-ylamino)-1-methyl-2-oxo-1,2-dihydropyrimido[4,5-d]pyrimidin-3(4 H)-yl)-4-methylphenyl)-3-(trifluoromethyl)benzamide; CAS 839707-37-8), Raf inhibitor BAY 43-9006 (4-[4-[[[[4-chloro-3-(trifluoromethyl)phenyl]amino]carbonyl]amino]phenoxy]-N-methyl-2-pyridinecarboxamide; CAS 284461-73-0), p38 MAP kinase inhibitor SB 202190 (4-[4-(4-fluorophenyl)-5-(4-pyridinyl)-1H-imidazol-2-yl]-phenol; CAS 152121-30-7), and Jnk MAP kinase inhibitor SP 600125 (anthra[1,9-cd]pyrazol-6(2H)-one; CAS 129-56-6). These findings indicate that interference with specific branches of TCR signaling, especially late phase kinases, can selectively affect individual hallmarks of T cell activation. In addition, it is likely that among the compounds interfering with CD69 upregulation but not caspase 3 activation are such small molecules that affect protein biosynthesis, sorting, and transport to the plasma membrane.

Importantly, our screen identified highly specific inhibitors of kinases that have not been previously shown to act in the TCR pathway. Examples of such compounds, shown in Fig. 3, are TWS119 (3-[[6-(3-aminophenyl)-7H-pyrrolo[2,3-d]pyrimidin-4-yl]oxy]-phenol; CAS 601514-19-6), an inhibitor of GSK3β^24^, and CCT018159 (4-[4-(2,3-dihydo-1,4-benzodioxin-6-yl)-5-methyl-1H-pyrazol-3-yl]-6-ethyl-1,3-benzenediol; CAS 171009-07-7), an inhibitor of the ATPase activity of Hsp90^25^. The compound TWS119 potently suppresses both caspase 3 activation and CD69 upregulation, whereas CCT018159 has no effect on caspase 3 but reduces the surface upregulation of CD69 in both CD4 and CD8 T cells.

### Lymphocyte activation assay

**Figure 4:**
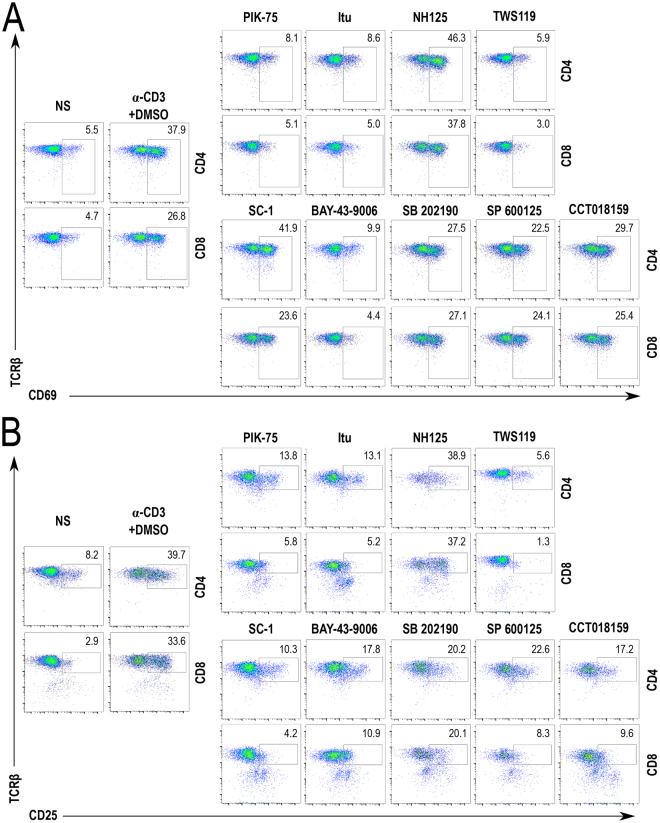
Identification of known and new inhibitors of lymphocyte activation among positive hits from the functional screen. (**A**) Flow cytometry plots showing the effects of selected inhibitors on CD69 upregulation in peripheral lymphocytes following a 3 h stimulation with plate bound anti-CD3 antibody. **(B)** Effects of same inhibitors on CD25 upregulation following an 18 h stimulation. Representative plots of the controls (NS and α-CD3 + DMSO) shown here were selected from a set of quadruplicates. Individual compounds were tested individually without replicates. NS: Non-stimulated samples (negative control); α-CD3 + DMSO: samples stimulated in the presence of DMSO (positive control).

We expanded our investigation to include a study of activation of peripheral T cells. Cells were isolated from lymph nodes of TCR-polyclonal B6 mice and tested for hallmarks of early and late activation following stimulation with plate-bound anti-CD3 antibody. Active caspase 3 staining which was highly relevant in the investigation of thymocyte reactivity was omitted here, as it is not a canonical outcome of mature peripheral T cell activation. We identified a largely identical subset of inhibitors that blocked the surface upregulation of the early activation marker CD69 (Fig. 4A) and late marker CD25 (Fig. 4B), but several compounds showed cell type-specific properties. For example, the compound NH-125 showed a mild suppressive effect on thymocyte activation, but did not inhibit lymphocyte activation, whereas PIK-75, Itu, and TWS119 were potent inhibitors in both cell types. The RasGRP1/Erk inhibitor Sc-1 affected CD25 but not CD69 upregulation. Two compounds, SB 202190 and SP 600125, had more pronounced effect on CD69 upregulation in CD4 T cells and thymocytes than in CD8 T cells. The screen therefore identifies compounds relevant to T cell receptor signaling in both differentiation and peripheral activation, and detailed analysis reveals cell type specificity and potential mechanism of action.

Our data indicate that the proposed strategy generates reliable hits in both thymocyte- and lymphocyte-based screens and can be successfully applied to the screening of compound libraries for small molecules affecting T cell receptor signaling. Even with the use of a comparatively small library of kinase inhibitors and were able to identify previously unexplored potential leads. Expansion of compound libraries to include other classes of enzyme inhibitors and other small molecules will likely yield more potential hits and contribute to our understanding of T cell receptor signaling and activation and pinpoint potential targets for pharmaceutic intervention. The strategy described here involves cell permeabilization and intracellular staining for active caspase 3. While this approach is feasible in relatively small screens, such as this one involving several 96-well plates per assay, its multistep nature, necessity of pelleting cells by centrifugation, and inevitable cell loss are not optimal for higher throughput screening, in particular in 384- and 1536-well formats. The assay can be adapted to such needs. For example, the use of cell-permeable fluorescent caspase sensors allows for sensitive detection of caspase activity and eliminates the need for cell permeabilization and multiple washing steps^17^.

### Data variability and assay robustness

**Figure 5:**
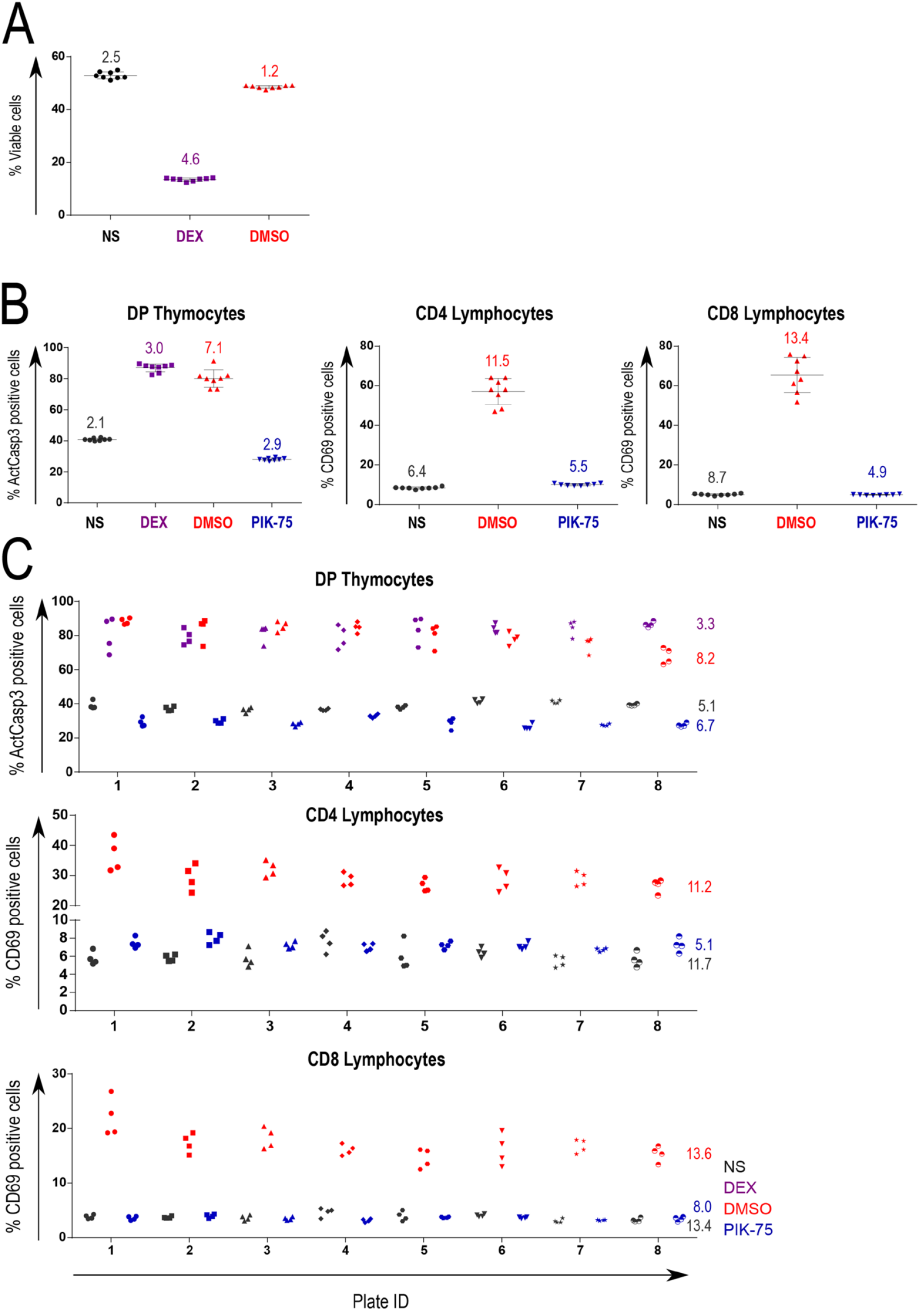
Reproducibility of screening assays. (**A**) Technical replicates for the positive (DEX: dexamethasone, purple symbols) and negative controls (NS: not stimulated, black symbols; and DMSO, red symbols) of the viability assay. Cells were assayed in eight replicates per condition by flow cytometry. The graph depicts primary data points, mean, and SD. Coefficients of variation (CV) are indicated for each data set. **(B)** Technical replicates for the positive and negative controls (NS: not stimulated, black symbols; DEX: dexamethasone, purple symbols; DMSO: TCR stimulation in the presence of DMSO, red symbols) and an assay compound (TCR stimulation in the presence of PIK-75; blue symbols), in thymocyte and lymphocyte assays. Caspase 3 activation in thymocytes (left panel) and CD69 upregulation in CD4 and CD8 lymphocytes (middle and right panel) were analyzed as in Figs 3 and 4. Cells were assayed in eight replicates per condition by flow cytometry. The graph depicts primary data points, mean, and SD. CV values are indicated for each data set. **(C)** Technical replicates for the positive and negative controls (NS: not stimulated, black symbols; DEX: dexamethasone, purple symbols; DMSO: TCR stimulation in the presence of DMSO, red symbols) and an assay compound (TCR stimulation in the presence of PIK-75; blue symbols), across individual plates. Cells were assayed in four replicates per plate per condition, in eight individual plates, by flow cytometry. CV values between plates is indicated for each data set.

We conducted a series of experiments to investigate data variation in both thymocyte- and lymphocyte-based assays. Figure 5A shows results of replicate testing in a thymocyte viability assay in the absence of TCR stimulation, designed identically to the experiment shown in Fig. 1B. The calculated coefficients of variation remained under 5% for all tested conditions. Figure 5B shows results of replicate testing in thymocyte specimens tested for caspase 3 activation and CD4 and CD8 lymphocytes tested for the upregulation of CD69 upon TCR stimulation in the presence or absence of one individual inhibitor, PIK-75. In all cases, the highest variation was observed in positive control samples, i.e. under conditions of uninhibited TCR stimulation, with CV values between 7–13%. Negative control (unstimulated) and strongly suppressed samples showed CV values between 2–9% in all tested cases. Figure 5C shows results of a cross-plate replication experiment in which thymocytes and lymphocytes were stimulated in eight individual plates, four wells per condition per plate. The observed cross-plate variation was generally higher than that within same plate, but remained under 15% in all tested conditions. Importantly, replicate testing, as well as all primary screens, was performed under non-HTS conditions (see Materials and Methods). We expect that the use of robot-assisted plate manipulation, including cell stimulation and staining, and high throughput flow cytometry instrumentation will result in increased reproducibility.

In conclusion, we propose a simple and robust strategy for the identification of small molecules affecting T cell receptor signaling and T cell activation. Other flow cytometry-based screening approaches have been previously proposed for the assessment of various aspects of peripheral activation of T cell subsets, for example the use of genetic fluorescent reporters to track T cell receptor signaling^26^, use of degranulation markers to follow activation of cytotoxic T cells^27,28^, and investigation of the activation state of individual signaling molecules^29^. Markers of apoptosis have been previously used to study cellular toxicity of compounds of interest specifically in immortalized T cell lines^30^. Our work expands the repertoire of tools that can be adapted for high-throughput screening.
